# Diagnosis value of ^18^F-Fluoro-d-glucose positron emission tomography-computed tomography in pulmonary hamartoma: a retrospective study and systematic review

**DOI:** 10.1186/s12880-023-00981-z

**Published:** 2023-02-07

**Authors:** Sheng Ye, Shen Meng, Shuang Bian, Cuicui Zhao, Jin Yang, Wei Lei

**Affiliations:** 1 Department of Pulmonary and Critical Care Medicine, The First Affiliated Hospital of Soochow University, No. 899 Ping Hai Road, Suzhou, 215006 Jiangsu China; 2 Department of Pulmonary and Critical Care Medicine, The Affiliated Hospital of Hangzhou Normal University, Hangzhou, 310015 Zhejiang China

**Keywords:** PET-CT, CT, Diagnosis, Pulmonary hamartoma, SUVmax

## Abstract

**Purpose:**

The diagnosis of pulmonary hamartoma (PH) based on computed tomography (CT) is a challenge, especially in patients with atypical imaging characteristics. This study was aimed at summarizing the imaging characteristic of ^18^F-Fluoro-d-glucose positron emission tomography-computed tomography (^18^F-FDG PET-CT) in PH and exploring the application value of PET-CT in the diagnosis of PH.

**Data and methods:**

Patients diagnosed with PH who had undergone PET-CT from literature pertaining were retrospectively analyzed, which were cases of publications from the Cochrane Library, PubMed, Excerpta Medica Database (EMBASE), China National Knowledge Infrastructure (CNKI) and Wanfang databases, from 2008 to June 2022. The other 20 cases of the collection were patients from our hospital from 2008 to June 2022. Patients’ symptoms, imaging characteristics of chest CT, PET-CT characteristics, the reason for PET-CT and the complications were analyzed.

**Results:**

In this retrospective study, a total of 216 patients were diagnosed with PH and had been examined by PET-CT. 20 of the cases were patients of our hospital from January 2008 to June 2022. The other cases were collected from the literature. The mean diameter of most PH lesions is 1.7 ± 1.0 cm. The mean maximum standardized uptake value (SUVmax) of the PH lesions was 1.2 ± 1.1. Most of their SUVmax were lower than internationally recognized cut-off value (SUVmax = 2.5). PET-CT was superior to CT in the diagnosis of PH but there was a correlation of between CT diagnosis and PET-CT diagnosis for the PH lesions. In order to draw the Receiver operating characteristic (ROC), we selected 29 patients with a clear SUVmax value of their PH lesion, and 29 lung cancer patients with clear SUVmax value in our hospital were collected as a control group. ROC curve analysis showed that the area under curve (AUC) of SUVmax was 0.899, and the optimal diagnostic threshold was SUVmax > 2.65. PET-CT could distinguish PH from malignant lesions with a sensitivity of 89.66% by applying a SUVmax of 2.65 as a cut-off in this study.

**Conclusion:**

PET-CT might be a useful tool to diagnose PH, which shows a better diagnostic sensitivity than CT. But PET-CT can not be used as a single diagnostic approach, which should be combined with other methods and the patients’ history to make the most correct diagnosis.

## Introduction

Pulmonary hamartoma (PH) is the most common benign lung tumor. When the popcorn calcification is present at the computed tomography (CT) scan, it usually suggests the diagnosis of PH [1]. Most PHs appear as solitary pulmonary nodules (SPN) and don’t need surgical resection. The benign diagnosis by CT helps guide the next step to formulate the time for reexamination and evaluating the lesion regularly. However, the typical findings can be seen in only 20% of all the cases [2], the atypical imaging characteristics make the diagnosis and differential diagnosis problematic. Sometimes it can be even misdiagnosed as lung cancer [3, 4].

Nowadays, the ^18^F-Fluoro-d-glucose positron emission tomography-computed tomography (^18^F-FDG PET-CT) plays an important role in the diagnosis, assessment of therapeutic effect and prognosis in SPN [5], inflammatory disorders [6] and rare diseases [7, 8]. It has been reported that PET-CT has a high accuracy for the detection and characterization of SPNs in lung diseases [9], the cut-off maximum standardized uptake values (SUVmax) of 2.5 is currently accepted [10]. When PH is diagnosed as a malignant or suspicious lesion by CT, PET-CT is feasible for further examination. However, PET-CT can only make a preliminary diagnosis, and the diagnostic gold standard still depends on pathology. At the same time, some PH with increased ^18^F-Fluoro-d-glucose (FDG) uptake could be identified malignant by PET-CT.

Up to now, only a few studies concentrated on the characteristics of PH in PET-CT and the diagnostic value of PET-CT in PH. Most of the literatures consider that the SUVmax of PH is low, manifested as low or no FDG uptake. However, there is no more suitable cut-off value to distinguish PH from pulmonary malignancy. Therefore, the goal of this retrospective study was to further explore the diagnostic value of PET-CT in PH, including the imaging characteristics of PH in PET-CT, the diagnostic sensitivity of PET-CT, and the cut-off value based on our data. In order to provide more basis for early and accurate diagnosis.

## Methods and materials

### Literature search and data collection

This retrospective study integrated the data of the single center and the patients who met the inclusion criteria in the literature. Publications regarding the use of ^18^F-FDG PET-CT in patients with PH were retrieved from the Cochrane Library, PubMed, Excerpta Medica Database (EMBASE), China National Knowledge Infrastructure (CNKI) and Wanfang databases, from 2008 to June 2022, using the terms of “pulmonary hamartoma”, “PH”, “positron emission tomography-computed tomography”, “PET-CT” and “PET/CT”. Duplicated references were excluded. Patients’ age, gender, clinical characteristics, chest CT findings, 18F-FDG PET-CT characteristics and the SUVmax of the PH lesions were extracted from each study for retrospective analysis. 20 involved patients were from The First Affiliated Hospital of Soochow University from January 2008 to June 2022. In addition, 29 lung cancer patients with SUVmax value in our hospital were collected to draw the ROC (receiver operating characteristic) curve as a control group. This retrospective single-center study was approved by the Ethics Committee of The First Affiliated Hospital of Soochow University, and the patient information was anonymized and de-identified prior to analysis. All methods were performed in accordance with the relevant guidelines and regulations.

### Image analysis

CT images and PET-CT images of 20 patients in our hospital were reviewed by 3 experienced radiologists and nuclear medicine specialists with > 10 years of experience blinded to the pathology. In case of conflict of opinions, 3 physicians will make diagnosis based on comprehensive analysis and complete discussion combined with relevant clinical data.

### Statistical analysis

All statistical analyses were performed using the SPSS® statistical software v. 25.0 (IBM Corp., New York, NY). Differences in proportions were assessed by χ^2^ test. *P* < 0.05 was considered statistically significant.

## Results

### Characteristics of the screened studies

A total of 216 patients confirmed to have PH and had undergone PET-CT were included in this study. A total of 10 eligible publications were retrieved [3, 4, 11–18], which involved 196 patients. And the rest 20 cases of the collection were patients from the First Affiliated Hospital of Soochow University. In order to draw the ROC curve, we enrolled 29 patients pathologically confirmed lung cancer from our hospital. Appropriate cases were selected for each analysis based on the differences in clinical data provided.

### Mean diameter and mean SUVmax analyzed in literature

A total of 165 eligible cases were included in this analysis, based on data from 4 articles [11–14]. All these cases were diagnosed PH pathologically. These 165 patients appeared as different whole in the 4 articles, which only provided the mean size and mean SUVmax, while the specific lesion size and SUVmax value of each patient were not provided. According to the table, the mean diameter of most PH lesions is 1.7 ± 1.0 cm. The mean SUVmax of the PH lesion was 1.2 ± 1.1, which means that most of their SUVmax values were lower than internationally recognized cut-off value (SUVmax = 2.5) [10], indicating low uptake or no uptake. It is consistent with the uptake characteristics of benign tumors.

### Comparison of diagnostic sensitivity between CT and PET-CT

In order to compare the diagnostic sensitivity between CT and PET-CT, a total of 84 eligible cases were included in this analysis. These 84 cases were provided with concurrent CT and PET-CT diagnosis, based on data of 64 patients from 7 articles [3, 4, 11, 13, 15–17] and 20 patients of our hospital, and all these cases had pathologic diagnosis of PH. There were significant differences between the two diagnostic methods (χ^2^ = 14.235, *P* < 0.001). The sensitivity of PET-CT was 78.6% (66/84); while CT has a sensitivity of 52.4% (44/84), indicating that PET-CT is superior to CT in the diagnosis of PH (Tables 1, 2).

**Table 1:** Mean diameter and mean SUVmax analyzed in researches

Group (literature)	Numbers of cases	Mean diameter (cm)	Mean SUVmax
1 [11]	16	1.6 ± 0.9	1.1 ± 0.5
2 [12]	32	2.1 ± 1.0	1.7 ± 0.9
3 [13]	12	1.4 ± 1.0	0.8 ± 0.7
4 [14]	105	1.6 ± 1.0	1.1 ± 1.2
Total	165	1.7 ± 1.0	1.2 ± 1.1

**Table 2:** Comparison of diagnostic sensitivity between CT and PET-CT

Diagnostic tool	Benign diagnosis	Malignant diagnosis	Total (literature)	Diagnostic sensitivity (%)	False negative rate (%)
CT	44	40	84 [3, 4, 11, 13, 15–17]	52.4	47.6
PET-CT	66	18	84 [3, 4, 11, 13, 15–17]	78.6	21.4

### Correlation between CT diagnosis and PET-CT diagnosis

In order to analyze the connection of diagnostic results between CT and PET-CT, a total of 84 eligible cases with concurrent CT and PET-CT diagnosis were included in this analysis, the inclusion criteria are the same as above. and all these cases had pathologic diagnoses of PH. As shown in Table 3, there was a specific correlation between CT diagnosis and PETCT diagnosis (χ^2^ = 4.671, *P* < 0.05). Among the 43 patients diagnosed benign by CT, the diagnostic sensitivity of PET-CT was 86.3% (38/44), but among the 40 patients diagnosed malignant by CT, the diagnostic sensitivity of PET-CT was only 70.0% (28/40). The results showed that there is a correlation of diagnostic sensitivity between CT and PET-CT, PET-CT showed a lower diagnostic sensitivity for PH lesions misdiagnosed as malignant by CT, which showed the limitation of PET-CT.

**Table 3:** Correlation between CT diagnosis and PET-CT diagnosis

PET-CT diagnosis	CT diagnosis		Total
	Benign	Malignant	
Benign	38	28	66
Malignant	6	12	18
Total	44	40	84

### Major imaging characteristics of CT in patients with PH

In order to analyze the imaging characteristics of CT of PH, we need to collect cases with detailed clinical data. A total of 25 eligible cases with CT characteristics and diagnosis were included in this analysis, based on data of 5 patients from 5 articles [3, 4, 15–17] and 20 patients from our hospital. All these cases had pathological confirmation of PH. These 25 patients had complete case data for detailed analysis. Detailed data were shown in Table 4. Except for one large lesion with a diameter of 180 mm, which was a pulmonary mass, other lesions ranged in size from 4 to 30 mm, which belonged to SPN. Five patients (5/25) had hamartoma lesions with calcification, 6 patients (6/25) had hamartoma lesions with fat density, and one patient had both calcification and fat density; none of the patients had typical popcorn calcification on imaging. Among all the cases, 3 patients were diagnosed benign (2 of which considered as hamartoma), 11 patients were diagnosed malignant, and the diagnosis of rest 11 cases was indeterminate. 8 patients underwent enhanced CT examination, and 4 of them showed enhancement in the CT images.

**Table 4:** Clinical manifestations and CT features of patients with pulmonary hamartoma

Patient (literature)	Age/Sex	Whether the patient had symptoms	Size (mm), Location	Whether the lesion has calcification	Whether the lesion has fat density	CT diagnosis	Whether enhanced in Enhanced CT
1	66/F	No	11, RML	No	No	Indeterminate	NA
2	68/M	No	14, LLL	No	No	Malignant	NA
3	50/F	Cough	9, RML	No	Yes	Benign	NA
4	36/M	No	10, RUL	Yes	Yes	Indeterminate	NA
5	69/M	Cough	12, RLL	No	No	Benign	No
6	67/F	No	11, RML	No	Yes	Indeterminate	NA
7	57/F	No	13, RLL	No	No	Indeterminate	NA
8	61/F	No	8, RML	No	No	Indeterminate	No
9	66/F	No	26, LUL	No	Yes	Malignant	NA
10	55/F	No	13, LUL	No	No	Indeterminate	No
11	72/F	No	15, LUL	No	No	Malignant	Yes
12	55/F	No	22, RLL	Yes	No	Malignant	Yes
13	50/M	No	16, LLL	No	No	Indeterminate	NA
14	70/F	No	8, LUL	No	Yes	Malignant	NA
15	64/M	No	8, RUL	No	No	Indeterminate	Yes
16	62/F	No	13, RUL	NA	No	Indeterminate	NA
17	42/M	Cough	15, LLL	No	Yes	Indeterminate	NA
18	30/F	No	22, LUL	No	No	Malignant	NA
19	47/F	No	21, LLL	No	No	Benign	NA
20	43/M	Cough	26, RLL	Yes	No	Malignant	NA
21 [15]	77/M	No	17, LUL	No	No	Indeterminate	Yes
22 [16]	30/M	Cough	NA, LUL, LML	Yes	No	Malignant	NA
23 [17]	35/M	Cough	180, Left lung	Yes	No	Malignant	NA
24 [3]	57/M	Cough	30, RML	NA	NA	Malignant	NA
25 [4]	29/M	Fever, Cough	13, LLL	No	No	Malignant	No

LLL, left lower lobe; LUL, left upper lobe; RLL, right lower lobe; RML, right medium lobe; RUL, right upper lobe; NA: not available; M: male; F: Female; SPN: small Solitary Pulmonary Nodule; PH: pulmonary hamartoma

### Major imaging characteristics of 18F-FDG PET-CT in patients with PH

**Fig. 1:**
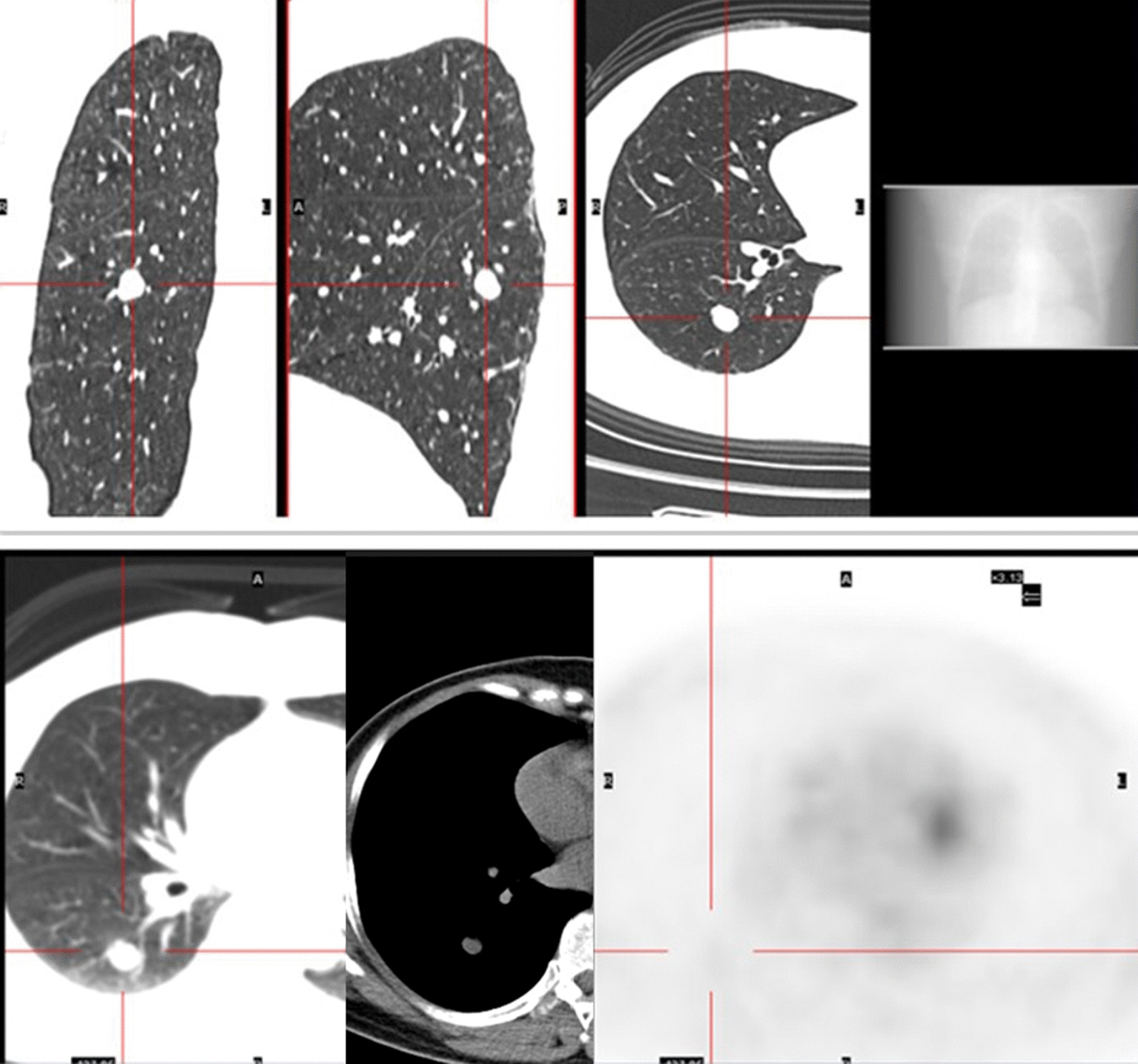
A kind of round nodular opacity was observed in the dorsal segment of the right lower lung, with a diameter of about 13 mm. No significant abnormal increase in FDG uptake was observed

A total of 25 eligible cases with PET-CT characteristics and diagnosis were included in this analysis, same as above. All these cases were diagnosed PH pathologically. Detailed data were shown in Table 5. PET-CT images of one case’s PH are shown in Fig. 1. All patients underwent PET-CT examination because the malignant possibility of the PH lesion could not be excluded. The cases included 2 patients combined with lung cancer, 1 patient combined with cervical cancer, which were confirmed by pathology. The results showed that only 2 patients had higher FDG uptake with a SUVmax of 3.0, which was higher than the recognized cut-off value. There were 5 cases combined with lymph node uptake. Of which 3 demonstrated submaxillary lymph node uptake, one demonstrated left cervical lymph node uptake combined with left upper lung cancer, one demonstrated right hilar lymph node uptake combined with right upper pulmonary mechanized pneumonia. No significant enlargement or increased FDG uptake of the mediastinal lymph nodes and rest hilar lymph nodes were observed.

**Table 5:** PET-CT features of patients with pulmonary hamartoma

Patients (literature)	Whether the lesion had high FDG uptake	Whether the lymph node had high FDG uptake	Whether other parts of the body had high FDG uptake	PET-CT diagnosis	Combined with other tumor or inflammation
1	No	No	No	Benign	Myoma of uterus
2	No	No	No	Malignant	No
3	No	No	No	Benign	No
4	No	No	Rectum wall	Benign	No
5	No	Right hilar lymph nodes	No	Benign	Mechanized pneumonia in RUL
6	No	No	No	Benign	No
7	No	Left submandibular lymph node	Nasal pharynx	Benign	No
8	No	Left submandibular lymph node	Nasal pharynx,	Benign	No
9	No	No	No	Benign	No
10	No	No	Cervix uteri	Malignant	Cervical cancer
11	No	No	No	Benign	No
12	No	No	No	Benign	Myoma of uterus
13	No	No	No	Malignant	No
14	No	No	Tumor in LLL	Malignant	Pulmonary melanoma in LLL
15	No	No	Tumor in RUL	Malignant	Lung cancer in RUL
16	No	No	No	Benign	No
17	No	No	SPN in RML, RLL. Nasal pharynx	Benign	Inflammation lesion in RML, RLL
18	No	Left cervical lymph node,	Right breast, BSO, left shoulder blade	Benign	Lung cancer in RLL
19	No	Submandibular lymph node	No	Benign	No
20	No	NA	NA	Malignant	No
21 [15]	No	No	No	Malignant	No
22 [16]	SUVmax:2.6	No	No	Malignant	No
23 [17]	SUVmax:3.0	NA	NA	Malignant	No
24 [3]	No	NA	NA	Malignant	No
25 [4]	No	No	Left upper femur	Malignant	No

LLL, left lower lobe; LUL, left upper lobe; RLL, right lower lobe; RML, right medium lobe; RUL, right upper lobe; NA: not available; SPN: small Solitary Pulmonary Nodule; PH: pulmonary hamartoma; BSO, bilterl slpingooophorectomy

On the whole, 14 cases were diagnosed as benign while 11 cases were diagnosed as malignant by means of PET-CT. Factors contributing to the diagnosis of malignancy included possible combination of malignant tumor or malignant tumor metastasis (3/11), multiple PH (1/11), giant PH (1/11), and purely misdiagnosis (6/11).

### The cut-off value we concluded based on our cases

**Fig. 2:**
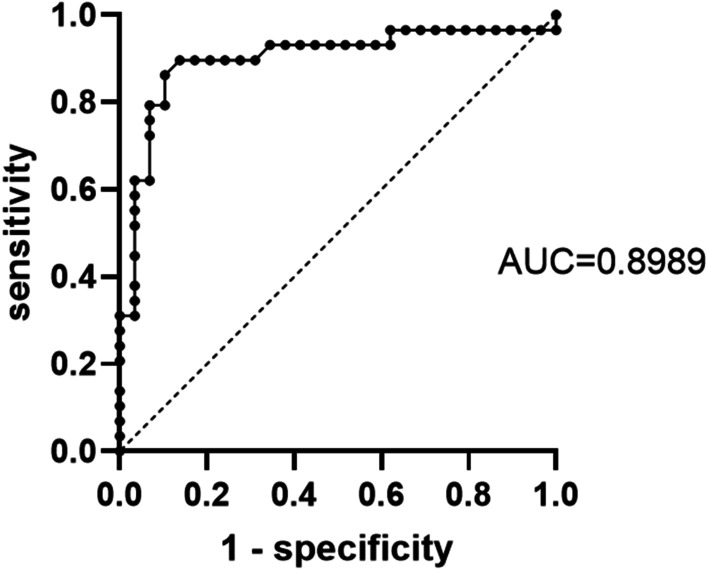
The ROC curve for SUVmax threshold to distinguishing malignant lesions from hamartoma lesions

A total of 29 eligible PH patients were included in this analysis, including 9 patients from literatures [3, 4, 12–18] and 20 in our hospital. The PH lesion of the 29 patients had clear SUVmax value for us to analysis and draw the ROC curve. In addition, 29 lung cancer patients with clear SUVmax value in our hospital were collected to draw the ROC curve as a control group. The SPN of the 29 patients was pathologically confirmed as early primary lung cancer after surgery, showing as single lesions on CT imaging. ROC curve analysis for discriminating malignant lesions and hamartoma lesions was performed. The ROC curve obtained was shown in Fig. 2. The area under the ROC curve (AUC) of SUVmax was 0.899 for malignant lesions and hamartoma lesions. The cutoff value of SUVmax, which distinguishing malignant lesions from hamartoma lesions was > 2.65 (Sensitivity 89.66%, specificity 86.21%), and the difference was statistically significant (*P* < 0.001). PET-CT could distinguish PH from malignant lesions with a sensitivity of 89.66% by applying a SUVmax of 2.65 as a cut-off in this study.

## Discussion

PH is usually asymptomatic, presenting as small, single, well-circumscribed round nodules [19]. However, typical findings might not be observed, thus CT findings cannot be diagnostic regarding malignancy [16, 17, 20]. At this point, PET-CT can be used for further evaluation. Like other benign lung lesion, PH usually shows a lower FDG uptake than malignant tumor. The SUVmax together with visual assessment improved the diagnostic accuracy [21].

First, a total of 165 cases were included in this analysis from 4 literatures, and the results are shown in Table 1. The SUVmax value of pulmonary hamartoma is lower than the internationally recognized cut-off value (SUVmax = 2.5) [10], indicating low uptake or no uptake. And the mean size of the PH lesion is 1.7 cm, which were in line with previous experience [22, 23].

We analyzed and compared the diagnostic differences between PET-CT and CT, and the results were shown in Table 2. In our study, the differences between the two diagnosis was statistically significant and the diagnostic sensitivity of PET-CT is superior to CT, which showed that PET-CT could be a valuable tool to evaluate PH with a higher diagnostic accuracy due to less misdiagnosed rate, which was consistent with the previous experience [21].

Based on the CT characteristics of the lesion, PET-CT further evaluate the lesions by combining the FDG uptake of the lesion and the surrounding lymph nodes [24]. In our study, we used PET/CT to evaluate the hilar and mediastinal lymph nodes in PH patients, the results were shown on Table 4. The upper results showing a higher diagnostic accuracy than CT. To explore the association between the two diagnostic modalities, we further analyzed the CT and PET-CT diagnosis of the 84 patients above. In our study, as shown in Table 3, there is a correlation of diagnostic sensitivity between CT and PET-CT, which showed that the diagnostic sensitivity of PET-CT was better than CT, but it still has certain limitations on atypical PH lesions.

Next, we analyzed 25 patients who had received PET-CT examination with detailed clinical data, and the results were shown in Table 4 and 5. PET-CT images of one case’s PH are shown in Fig. 1.

First, we analyzed the CT characteristics of PH lesions. Case 23 was a patient with a giant PH, the diameter of the lesion was up to 18 cm, which was a lung mass. And the other PH lesion of the collection belonged to SPN. Among the 25 patients with PH, most of the patients lacked calcification (23/25), fat density (22/25), and no patients showed popcorn calcification. Atypical CT imaging findings lead to unclear CT diagnosis; Only 2 cases were diagnosed as PH by CT. 9 patients underwent contrast-enhanced CT, and 4 of which showed enhanced images. Christensen et al. showed that PET-CT is preferable to enhanced CT in evaluating indeterminate pulmonary nodules [25].

PET-CT examination was performed in all cases because of the lack of typical PH characteristics and a malignant diagnosis could not be ruled out. According to the results of PET-CT examination, most PH lesions (23/25) and lymph node showed no significant increase in FDG uptake, which was consistent with a benign lesion. In our study, 3 cases were diagnosed as metastatic lung cancer lesion because of the combination of malignant tumor. Because metastatic lung cancer cannot be ruled out, PETCT could make a malignant diagnosis. This result has not been mentioned in previous studies [11–14, 18].

Based on the above results, we analyzed the factors influencing the diagnostic sensitivity of PET-CT in PH. The factors contributed to diagnosis of PH by means of PET-CT including a single lesion, low SUVmax of lesion (lower than the internationally recognized cut-off value of 2.5) and no other combined malignant tumor. On the contrary, some PH lesions have increased FDG uptake (which may be caused by the large size of the lesions, combined with inflammation, etc.) could cause false negative, which was consistent with the literature [26]. Meantime, according to our results, when patients complicated with other malignant tumors, PH lesions could be misdiagnosed as metastatic malignant nodules. In this situation, the malignant diagnosis of PET-CT is reasonable, but the possibility of combination of benign neoplasms, such as PH, should be considered.

The ability of PET-CT to differentiate benign lesions from malignant is significant for the following clinical decisions. Studies have been performed to examine the accuracy of the cut-off value of 2.5. Aleksandar et al. [27]hold the view that an SUV below 1.25 was always combined with benign lesions. In our study, we obtained one SUVmax cut-off value of 2.65 according to the ROC curve, which was higher than the previous experiences [27–29]. Several possible reasons should be taken into consideration. First, the SUV is affected by a large number of technological factors (inter scanner variability, image reconstruction method, etc.) and biological factors (serum glucose level, body weight, etc.) [30]. As PET-CT becomes more widely used in the evaluation of non-malignant tumors and the number of relevant studies has increased, the cut-off value is constantly changing. Second, the cases included to plot the ROC curve were not sufficient enough to be convictive, causing selection bias. Meanwhile, Giant PH [17], multiple PH [16] and PH combined with pneumonia [31] can demonstrated a higher uptake could make the cut-off value increasing. Last, the SUVmax doesn’t take the volume of the tumor into account, this may be a significant limitation because tumor volume could affect the SUVmax, which may affect the results of the study [32]. However, Sim et al. even thought that using threshold SUVmax values to differentiate malignant from benign lesions is unrealistic [24]. Meantime, in our study, other factors should also be considered, including FDG uptake in mediastinal and hilar lymph nodes, the patients’ co-morbidities and nuclear medicine physician experience. In summary, SUVmax cannot be considered as a single diagnostic factor.

There are some limitations to our study. First, our systematic review was based on published studies, potential publication bias may have been present. Second, since there are few literatures on the value of PET-CT in the diagnosis of PH, so most of the literatures we cite are the value of PET-CT in the diagnosis of SPN, which means that our default PH belongs to a kind of SPN. Last, the number of patients is relatively small and these results will need to be verified in a large sample size.

## Conclusion

In conclusion, our study showed that PET-CT might be a useful tool in the diagnosis of PH. The diagnostic sensitivity of PET-CT has been improved than CT, but it still has certain limitations on atypical PH lesions. The PET-CT diagnosis and SUVmax value may help patients with indeterminate SPNs with a better selection for surgical resection and radiological follow-up. But PET-CT can not be used as a single diagnostic approach, which should be combined with other methods and the patients’ history to make the most correct diagnosis.

## Data Availability

All data generated or analyzed during this study are included in this published article.

## Abbreviations

PH: Pulmonary hamartoma
CT: Computed tomography
^18^F-FDG PET-CT: ^18^F-Fluoro-d-glucose positron emission tomography-computed tomography
SPN: Small pulmonary nodules
SUVmax: Maximum standardized uptake values
ROC: Receiver operating characteristic
